# Aberrant DNA Methylation and Prostate Cancer

**DOI:** 10.2174/138920211797904061

**Published:** 2011-11

**Authors:** Sunipa Majumdar, Eric Buckles, John Estrada, Shahriar Koochekpour

**Affiliations:** 1 Stanley S. Scott Cancer Center, School of Medicine, Louisiana State University Health Sciences Center, New Orleans, LA 70122, USA; 2 Department of Biology, Dillard University, New Orleans, LA 70122, USA; 3 Departments of Cancer Genetics and Urology, Roswell Park Cancer Institute, Elm & Carlton Streets, Buffalo, New York 14263, USA

**Keywords:** Epigenetics, Genome, Methylation, Prostate cancer.

## Abstract

Prostate cancer (PCa) is the most prevalent cancer, a significant contributor to morbidity and a leading cause of cancer-related death in men in Western industrialized countries. In contrast to genetic changes that vary among individual cases, somatic epigenetic alterations are early and highly consistent events. Epigenetics encompasses several different phenomena, such as DNA methylation, histone modifications, RNA interference, and genomic imprinting. Epigenetic processes regulate gene expression and can change malignancy-associated phenotypes such as growth, migration, invasion, or angiogenesis. Methylations of certain genes are associated with PCa progression. Compared to normal prostate tissues, several hypermethylated genes have also been identified in benign prostate hyperplasia, which suggests a role for aberrant methylation in this growth dysfunction. Global and gene-specific DNA methylation could be affected by environmental and dietary factors. Among other epigenetic changes, aberrant DNA methylation might have a great potential as diagnostic or prognostic marker for PCa and could be tested in tumor tissues and various body fluids (e.g., serum, urine). The DNA methylation markers are simple in nature, have high sensitivity, and could be detected either quantitatively or qualitatively. Availability of genome-wide screening methodologies also allows the identification of epigenetic signatures in high throughput population studies. Unlike irreversible genetic changes, epigenetic alterations are reversible and could be used for PCa targeted therapies.

## 1 INTRODUCTION

Prostate cancer (PCa) is the most frequently diagnosed non-skin malignancy and the third leading cause of cancer-related death in men in the Western world [1]. PCa is one of the most complicated human tumors and, like many other malignancies, arises from progressive genetic and epigenetic alterations. The field of epigenetics has rapidly evolved and influenced research in different biological phenotypes such as aging, memory formation, and embryological development. Overall, epigenetic defects reported in cancers include reactivation of embryonic genes, loss of imprinted genes changing inactive and active alleles, dysregulated expression of micro-RNAs, increased gene recombination, and transcriptional silencing of tumor-suppressor and housekeeping genes. Epigenetics encompasses several different phenomena, such as DNA methylation, histone modifications, RNA interference, and genomic imprinting [2]. Epigenetic processes regulate gene expression and can change malignancy-associated phenotypes such as growth, migration, invasion, or angiogenesis. 

Unlike many other genetic changes, epigenetic processes are reversible and do not affect DNA sequence or quantity. However, they promote genomic instability that might lead to oncogenic activation and / or inactivation of tumor suppressors. Normal methylation levels of various genes preserve cellular homeostasis. Among all recognized epigenetic alterations, aberrant DNA methylation (hypo- and hypermethylation) is the most important and the best characterized change in PCa [3]. Hypermethylated genes in PCa include DNA damage repair genes (e.g., glutathione *S*-transferase Pi (GSTP1) and the DNA alkyl-repair gene O^6^-methylguanine DNA methyltransferase (MGMT)), hormonal response genes (e.g., androgen receptor (AR), estrogen receptor (ER)), cell-cycle control genes (e.g., CDKN2A), tumor-suppressor genes (e.g., *VHL, RB1, APC*), apoptosis genes (e.g., death-associated protein kinase (DAPK)), and invasion and metastasis genes (e.g., Cadherins, CD44, TIMPs). In addition, global and gene-specific hypomethylation have also been associated with PCa. Epigenetic events may also play a role in benign prostatic hyperplasia (BPH). Epigenetic regulatory mechanisms appear very sensitive to external stimuli or influences such as diet and oxidative stress. A comprehensive review on epigenetic alterations in PCa is beyond the scope of this review. Our intention is to provide a synopsis of widely known DNA methylated genes and their biological activity and pathways, interaction between DNA methylation and histone modification, epigenetic biomarkers for PCa diagnosis and prognosis, and epigenetic targets for PCa therapy. Finally, we review a number of methods for detection of DNA methylation and available data bases and computational analytical tools. 

## 2 DNA METHYLATION AND REGULATION OF GENE EXPRESSION

Epigenetic mechanisms such as DNA methylation and histone modification play an essential role in many molecular and cellular alterations associated with the development and progression of prostate cancer [4, 5]. In mammalian cells, most of the chromatin exists in a condensed, transcriptionally silenced form called hetero-chromatin. Euchromatin is less condensed and contains most of the actively transcribed genes. Histones and DNA are chemically modified with epigenetic markers that influence the chromatin structure by altering the electrostatic nature of the chromatin or by altering the affinity of chromatin binding proteins. DNA methylation is usually associated with histone deacetylation, chromatin condensation, and gene silencing [5-7].

DNA methylation leads to gene-silencing either by inhibiting the access of target binding sites to the transcriptional activators [8] or by promoting the binding of methyl-binding domain proteins, which can mediate repression through interaction with histone deacetylases (HDACs) that promote chromatin condensation into transcriptionally repressive conformations [9, 10].

DNA methylation involves the addition of a methyl group to the fifth carbon position of the cytosine pyrimidine ring *via* a methyltransferase. DNA methylation refers to the covalent bonding of a methyl group specifically to the dinucleotide CpG, which is catalyzed by the family of enzymes known as DNA methyltransferases (DNMTs). It is thought that DNA methylation alters chromosome structure and defines regions for transcriptional regulation. This covalent modification of multiple DNA sites by methylation is a heritable and reversible epigenetic process, which is involved in the regulation of a diverse range of biological processes [11-13]. Clusters of CpG sites are dispersed around the genome and are referred to as CpG islands, stretches of DNA ranging from 0.5 to 5 kb with a guanine-cytosine (GC) content of at least 50%. These islands are found in the promoter region of about 60% of genes, in exons and introns, and in repetitive elements. Most CpG islands in the promoter regions are unmethylated, whereas CpG islands in intronic regions and repetitive sequences are heavily methylated, perhaps to help the cell identify regions for gene transcription [14].

There are two distinct classes of DNMTs. The first class consists of *de novo* methyltransferase**s **(DNMT3a and DNMT3b) that methylate DNA irrespective of whether the template is hemi-methylated or not. These enzymes are involved in the establishment of new DNA methylation patterns during development. The maintenance DNA methyltransferase DNMT1 belongs to the second class of enzymes. Disruption of the mouse *Dnmt1 *gene results in genome-wide demethylation and developmental arrest. Thus, the role of DNMT1 in propagating parental DNA methylation during replication cannot be substituted by other DNMTs [15]. Without the DNMTs, the replication machinery itself would produce daughter strands that are unmethylated and, over time, would lead to passive demethylation.

Outside of CpG islands, CpG methylation is thought to suppress the transcription of transposable elements and spurious initiation of transcription elsewhere. DNA methylation abnormalities, such as the gain of methylation in normally unmethylated promoters or other regulatory regions (hypermethylation), contribute to tumorigenesis by decreasing the activity of tumor-suppressor genes. Loss of methylation in normally methylated repetitive sequences that lead to activation of proto-oncogenes and genomic instability is evident in almost all human tumor types [16,17]. DNA methylation is the best established epigenetic mark that is critical for the allele-specific expression of imprinted genes [18]. Hypomethylation of specific chromosomal domains has been linked to chromosomal instability [19]. Chromosomal abnormalities associated with hypomethylation include isochromosomes, unbalanced juxtacentromeric translocations, and whole-arm deletions. DNA hypomethylation of repetitive elements, retrotransposons, and CpG poor promoter regions plays an important role in tumorigenesis [20]. Hypomethylation of repetitive sequences and retrotransposons are associated with chromosomal rearrangements and translocation to other genomic regions, thereby promoting genomic instability [21, 22].

CpGs are underrepresented in mammalian genomes and occur in only 1% of the genome, lower than the expected statistical fraction of 6%. The methyl donor for this reaction is supplied by S-Adenosyl Methionine (SAM). This substrate is recycled through a folate- and cobalamin-dependent pathway. Hypomethylation or loss of methylation can be accelerated by altering this regenerative process through a deficiency of Vitamin B_9_ (folate), vitamin B_12_, or other substrates [23]. There are multiple roles for DNA methylation in mammals and disruption of this process during early development by the inactivating DNMTs is lethal [24]. 

DNA methylation also has a putative role in genome defense. All cells in culture or organisms in environment challenged life are subject to stringent conditions of selection, even the cell which has been forced by its innate recombination mechanisms to tolerate the genomic insertion of foreign DNA can avail itself of an ancient defense mechanism against the genetic activity of foreign DNA that could carry active genes. Since promoter methylation has been identified as part of a mechanism for the long-term silencing of genes and DNA segments, the *de novo *methylation of integrated foreign DNA can be contemplated as a defense mechanism or at least as an integral part of it [25].

Methylation is also involved with genomic organization and silencing unneeded genes in differentiated cells. During this process, DNA methylation acts as a stable tag on the promoter of a gene that recruits methyl-binding proteins (MBPs) and other proteins such as HDACs, to form large-scale heterochromatic structures that silence the associated genes. Changes in DNA methylation during cancer formation have seemingly divergent effects on the cell. 

**Fig. (1):**
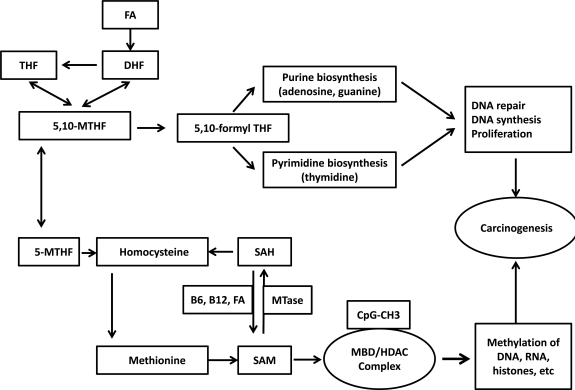
**Schematic representation of dietary factors, one-carbon metabolism, methionine cycle in DNA methylation.** One-carbon metabolism is the best understood pathway of dietary regulation of DNA methylation. Folic acid is converted to dihydroflate (DHF) and then to tetrahydrofolate and finally to methylene tetrahydrofolate (MTHF). 5, 10-MTHF is required for the synthesis of nucelic acids, and 5- MTHF is required for the formation of methionine from homocysteine *via* folate- and B12-dependent methionine synthase reaction. Methionine adenosyl transferase transfers adenosine to methionine and generates S-adenosylmethionine (SAM), which is the main methyl donor. Subsequently, SAM is converted to S-adenosyl homocysteine (SAH), which has high binding affinity to methyltransferases (MTase). A dietary supply of vitamins B12, B6, and folic acid *via* several steps regenerate SAH to SAM. Methylene tetrahydrofolate (MTHF) can direct folic acid (FA) to nucleotide synthesis as an important path for DNA synthesis, cell growth, and DNA repair or for conversion of homocysteine to methionine. DNA-methyl transferases (MTase) methylates the CpG island which recruits the methyl binding domain (MBD) and histone deacetylases (HDACs) to the methylated DNA and leads to histone deacetylation, condensation of chromatin, loss of transcription factor binding, and silencing of the gene expression in cancer and other premalignant conditions.

Three types of altered DNA methylation patterns have been observed in human tumors: hypomethylation, hypermethylation, and loss of imprinting [26]. DNA hypomethylation occurs in many tumors, particularly in advanced stages, and is generally assumed to be a genome-wide event [27, 28]. By comparison, DNA hypermethylation occurs at specific regulatory sites, such as in the promoter regions of tumor-suppressor genes, and thereby decreases the expression of individual genes [29-31]. As a consequence, hypermethylation may be functionally equivalent to an inactivating gene mutation. Loss of imprinting, which refers to the loss of differential expression of the parental alleles, is often seen in embryonal tumors [32, 33]. While genome-wide hypomethylation, which could lead to activation of previously silenced genes, is seen in some advanced, metastatic PCas [28], most studies have emphasized DNA hypermethylation as an important mechanism for inactivation of key regulatory genes in PCas [27, 34]. For example, decreased expression of E-Cadherin, a component of the E-Cadherin/catenin cell adhesion complex, is associated with poorly differentiated and late-stage PCa and is closely linked to the progression of the disease [35]. Since the promoter of E-Cadherin is frequently found to be hypermethylated in prostate tumors and PCa cell lines and can be reversed by demethylation, it is likely that hypermethylation accounts for the reduced expression [31]. In contrast, hypermethylation is not responsible for the observed reduction in PCa of P-Cadherin expression, another member of the Cadherin family [36], illustrating the gene selectivity of DNA hypermethylation (Fig. **1**). 

## 3 HYPERMETHYLATED GENES IN PROSTATE CANCER

**Fig. (2):**
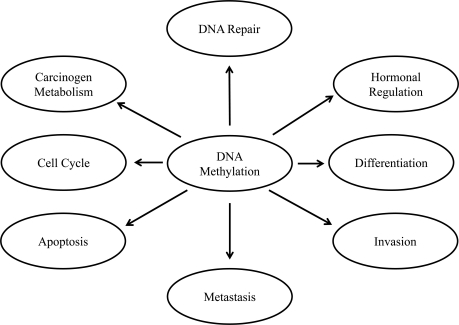
**Schematic diagram of involvement of DNA methylation in different cellular processes.** DNA methylation is the best known epigenetics alteration in prostate cancer. DNA methylation can regulate gene expression and can function in favor of malignancy-associated phenotypes such as cellular differentiation, growth, migration and invasion, metastasis, apoptosis, hormonal regulation of steroid receptors, and DNA repair. DNA methylation appears to be very sensitive to external stimuli or influences such as diet and oxidative stress.

DNA hypermethylation is the most common and best-characterized epigenetic abnormality in human malignancies, including PCa. Significantly, many of the affected genes encode proteins that are involved in critical cellular processes and/or have tumor-suppressor activity (Table **1**). Pathways frequently disrupted by CpG island hypermethylation include DNA damage repair, hormonal responses, tumor-cell invasion/metastasis, and cell cycle control (Fig. **2**). For many of these genes, promoter hypermethylation is often the primary or only mechanism underlying functional loss in PCa. Inappropriate silencing of these genes can contribute to cancer initiation, progression, and metastasis. Some of the hypermethylation occurs in the early stages of PCa progression; along the multistep process of prostate carcinogenesis; some correlate with pathological grade or clinical stages of PCa; some contribute to invasiveness, metastasis and androgen independence of PCa [37]. 

### 3.1 DNA Damage Repair Genes 

DNA repair is a correcting mechanism that maintains genomic integrity during replication or after DNA damage. Cells that are defective in the components of DNA repair pathways exhibit higher rates of spontaneous DNA mutations, which can lead to cancer [38]. The hypermethylation of two genes involved in DNA damage repair, the detoxifier gene glutathione *S*-transferase Pi (GSTP1) and the DNA alkyl-repair gene O^6^-methylguanine DNA methyltransferase (MGMT), has been reported in PCa. 

Glutathione S-transferase (GST) is a family of enzymes involved in the detoxification of xenobiotics and oxygen radicals [39]. Human GSTs are classified into distinct families; five of which encode cytosolic GSTs, α, µ, π, σ, θ forms. The product of π gene (GSTP1), which is located on 11q13, can detoxify environmental electrophilic carcinogens and oxidants and might have a genomic care taker role by preventing oxidant and electrophilic DNA damage and the resulting mutations [40]. GSTP1 expression is detectable in normal tissues at varying levels in different cell types. Notably, a loss or low level of GSTP1 expression has been reported in high-grade prostatic intraepithelial neoplasia (PIN) and PCa. Methylation of the GSTP1 gene promoter has been the most frequently detected epigenetic alteration, and occurs in over 90% of cancerous samples and about 70% of prostatic intraepithelial neoplasia (PIN) samples [41, 42], but is rarely detected in normal prostate or benign prostatic hyperplasia (BPH) tissues. It is also detected in a subset of proliferative inflammatory atrophy lesions (PIA). At the present time, decreased GSTP1 expression is the most common epigenetic alteration in PCa. 

MGMT is a DNA repair protein that removes mutagenic and cytotoxic alkyl adducts from genomic DNA. Tumors that lack MGMT expression have a higher incidence of point mutations in the genes encoding p53 and K-*ras, *which may influence cancer progression. The MGMT promoter contains a CpG island that is methylated in many human cancers [43]. Over- expression of MGMT reduces the risk of mutations after exposure to methylating agents while loss is associated with increased sensitivity to methylating agents and carcinogenicity. Results from studies evaluating the MGMT promoter methylation in PCa have been unequivocal, with moderate to high levels of methylation detected in some studies [44, 45], but not others [46, 47]. Further work will be necessary to resolve this discrepancy. 

### 3.2 Hormonal Response Genes 

The prostate is an endocrine gland that responds to sex hormones such as androgens, estrogens, and progesterones through their specific receptors. These agents bind to specific cellular receptors to mediate their physiological effects. 

#### 3.2.1 Androgen Receptor

Testosterone and 5α-dihydrotestosterone are the most important male hormones, with actions mediated through the androgen receptor (AR), resulting in the development and maintenance of the prostate [48]. The AR gene is located on chromosome Xq11–12. The 5’ region of the AR gene contains a CpG island according to the criteria established by Gardiner-Garden and Frommer [49] indicating that it might be regulated by DNA methylation. Jarrard *et al.* [50] analyzed the influence of AR promoter methylation on AR gene expression in PCa cell lines. Genetic alterations that alter the sensitivity of the AR to androgens, including AR gene mutations [51] and amplifications [52] without loss of AR expression, are thought to play a key role in the development of hormone-independent advanced PCa. Epigenetic changes have been accounted for alterations in the AR expression in 28% of hormone-independent PCa. Epigenetic regulation, including CpG methylation and histone acetylation, play important roles in the regulation of AR [53]; however, the frequency of AR methylation appears to be low in PCa [54-56]. 

#### 3.2.2 Estrogen Receptors

Estrogens, which have been used for the treatment of PCa for decades [57], require estrogen receptors (ERs) to mediate their activity [58-60]. The prostate expresses two types of estrogen receptors: ERα (ER1) and ERβ (ER2) [61]. Lost or decreased expression of ER1 and ER2 in PCa has been documented [62-65]. The ER1 gene is frequently methylated in PCa and methylation status is associated with tumor progression [66]. The ER2 promoter contains a typical CpG island [67]. Hypermethylation of the ER2 proximal promoter in the PCa cell lines and high frequency in human PCa has been documented [56, 68, 69]. Methylation of the ER1 and ER2 gene promoters is detectable in BPH; however, the extent of ER1 and ER2 promoter methylation is significantly less in the BPH samples than in prostatic tumors, indicating that prostate carcinogenesis induces ER gene hypermethylation [66, 69]. 

#### 3.2.3 Retinoic Acid Receptors

Retinoids, a group of natural and synthetic vitamin A analogs, are important factors in modulating cell growth, differentiation and apoptosis which have been shown to suppress carcinogenesis *in vitro* and *in vivo*. The retinoids are among the most investigated classes of chemo-preventive drugs for PCa. Their effects are primarily mediated through two classes of nuclear receptors – retinoic acid receptors (RARs) and retinoid X receptors (RXRs). These classes belong to the steroid/thyroid hormone – receptor superfamily and are composed of three subtypes (α, β, γ) [70]. The RARβ gene has two different promoters and expresses RNA transcripts that undergo alternative splicing [71]. The RARβ2 promoter contains a CpG island that is aberrantly methylated in PCa [72]. In PCa, RARβ2 expression is also controlled epigenetically, and a high frequency of aberrant methylation has been noted in clinical samples [47,54, 68 70, 73-77]. RAR β2 gene methylation is a frequent event in PCa. In addition, the RAR β2 promoter is methylated in 20% of PIN samples, a putative PCa precursor. Therefore, RAR β2 gene methylation appears to occur early in PCa etiology, and is implicated in cancer initiation [46, 72]. 

### 3.3 Cell Cycle Control Genes

A distinguishing characteristic of tumor cells is uncontrolled growth, which is regulated by the cell cycle pathway. Many genes act as checkpoints that regulate the cell cycle and if these genes become defective, may lead to carcinogenesis and progression of PCa [78]. 

The tumor-suppressor gene p16/CDKN2 is one of the cyclin-dependent kinase inhibitors (CDKIs). CDKN2A, a key protein in the signaling pathway, can be affected by a variety of genetic and epigenetic changes, including hypermethylation in PCa. Aberrant CDKI expression is observed in many tumor tissues, including the prostate [45, 79, 80]. Results regarding the frequency of CDKN2A promoter methylation are inconsistent in prostate tumors, ranging from 3% to 77%; other studies have investigated the role of hypermethylated CDKN2A in the carcinogenesis and progression of PCa [45, 47, 79-85]. Herman *et al.* first reported that inactivation of CDKN2A by DNA methylation in PCa [86]. The CDKN2A gene was frequently methylated in tumor tissue (77%). These data support p16 methylation as a potential biomarker for early detection of PCa. Other CDKIs such as p14, p21, p27 and RB1 are rarely methylated in prostate tumors, and thus, are probably not good candidates as biomarkers [45].

The RAS family of proto-oncogenes plays a key role in the signal transduction pathways involved in cellular proliferation and survival, interacting with other regulatory circuits of cell growth and death. RAS association domain family protein 1 isoform A (RASSF1A) is known as a tumor suppressor gene. The RASSF1A protein was found to be associated with the DNA repair protein and to mediate the apoptotic effect of oncogenic Ras [87, 88]. Inactivation of RASSF1A may result in dysregulation of DNA repair system and the Ras-dependent growth control in cancer cells. The RASSF1A gene is silenced by aberrant methylation of the promoter in a large fraction of various cancers including prostate [89]. In prostate tumors, RASSF1A promoter methylation is a common event, occurring in 49% to 99% of tumor tissues and it has been shown to be associated with aggressive PCa [44, 47, 89].

### 3.4 Tumor-Suppressor Genes

Function loss of classic tumor-suppressor genes through DNA hypermethylation is not a common event in PCa. For instance, methylation of RB1 [90], hMLH1 [91], and VHL [30] has been frequently detected in other types of cancer, but not in PCa. Methylation inactivation in CDKN2A and CDH1 has shown moderate to high prevalence in PCa [82, 86, 92]. 

The adenomous polyposis coli (APC) gene, located on chromosome 5q21-22, is known as tumor-suppressor gene that is responsible for Familial Adenomatous Polyposis (FAP). FAP is frequently identified in colorectal cancers, but not in PCa [93, 94]; however, involvement of APC hypermethylation has been described in different studies [44, 47, 75, 79, 81, 95, 96]. β-catenin mutations have been detected in various tumors and are relatively rare in PCa [94]. However involvement of γ - catenin methylation in prostatic carcinogenesis has been recently documented [97]. 

Additional genes with putative tumor-suppressor function undergoing epigenetic inactivation by hypermethylation in PCa include KAI1 (a prostate-specific tumor metastasis suppressor gene) [98], inhibin-α (a member of the transforming growth factor–β family of growth and differentiation factors) [99], and DAB2IP, a novel GTPase-activating protein for modulating the Ras-mediated signaling pathway [100]. It is unknown, however, whether hypermethylation of these genes plays a role in prostate carcinogenesis or has a role as a biomarker for PCa diagnosis.

### 3.5 Apoptosis Genes

Programmed cell death (apoptosis) is a critical process for carcinogenesis in human. Typical morphological characteristics of apoptosis include damage to the plasma membrane, condensation and fragmentation of the nucleus, and DNA fragmentation [101]. A major component of the apoptosis pathway is the caspase family. However, other genes, including death-associated protein kinase (DAPK), fragile histidine triad (FHIT), solute carrier family 5A8 (SLC5A8), vesicular monoamine transporter 2 (SLC18A2), and tumor necrosis factor receptor superfamily, member 10C (TNFRSF10C) are also involved in this pathway. A repressed expression of these genes by hypermethylation in the promoter region has been shown for PCa [46, 47, 81, 102-106]. However, DAPK and FHIT may have a limited value due to a persistently low frequency of methylation in tumors and normal tissues [46, 47, 81, 101]. SLC5A8, SLC18A2, and TNFRSF10C were found to be hyper-methylated in 50% to 88% of PCa and were significantly downregulated in the tumor when compared to normal prostate tissues [103-105, 107-109]. It is noteworthy that, the expression of SLC18A2 and TNFRSF10C is inversely associated with biochemical recurrence after radical prostatectomy [110]. 

### 3.6 Invasion and Metastasis Genes

Metastasis is an extremely complicated process that occurs through a series of sequential steps involving invasion, transport, adhesion to distant sites, and outgrowth into a secondary organ. Although metastases are the cause of 90% of human cancer deaths, little is known about the genetic and biochemical determinants of metastasis. Cell–cell adhesion and cell–substrate adhesion are critical to the preservation of the normal tissue architecture. These phenotypes are regulated by a group of proteins known as cell adhesion molecules (CAMs). Cadherins are a large family of CAMs that are involved in cell–cell adhesion. Disruption of the cell adhesion system can lead to tumor infiltration and metastasis [111,112]. 

E-Cadherin (CDH1), an important member of the Cadherin family of cell adhesion molecules, is a transmembrane glycoprotein whose extracellular domain mediates cell-cell adhesion through calcium-dependent homophilic interaction [113]. Decreased CDH1 expression is observed in many cancers, a significant correlation between loss of CDH1 expression and invasion and metastasis has been documented. With regard to PCa, correlations between decreased CDH1 expression and prognostic factors including tumor grade, stage and ploidy, have been reported [114, 115]. Methylation of the CDH1 promoter region has been detected in PCa cell lines [92]. In human prostate tumors, expression of CDH1 is strongly reduced and its promoter is methylated to varying degrees [44, 74, 92, 116]. The 5’ CpG island of CDH1 is densely methylated in PCa cell lines [31]. 

Matrix metalloproteinases (MMPs) are proteolytic enzymes that degrade the extracellular matrix and the basement membrane. High expressions of these enzymes have been associated with tumor growth, invasion, and tumor-induced angiogenesis [117]. These pathways are controlled by the balance between the levels of the MMPs and tissue inhibitors of metalloproteinases (TIMPs) [118]. TIMP-1, TIMP-2, and TIMP-3 are widely investigated members of this family involved in tumor progression and metastasis in a variety of human cancers. TIMP-2 expression appears to have a tumor-promoting role in PCa [119]. The promoter region of TIMP-3 was found to be methylated in 97% of prostate tumors [79] and two studies found TIMP-3 promoter methylation in 37% and 41% of urine sediments from PCa patients [84, 102]. 

CD44 is an integral membrane protein that is involved in matrix adhesion and signal transduction. CD44 is classified as a metastasis suppressor because decreased CD44 expression is associated with the progression of PCa to a metastatic state [120]. It has been reported that loss of standard CD44 expression in PCa predicts a poor prognosis [121, 122]. The methylation of the 5’ CpG island of CD44 is associated with transcriptional inactivation in PCa cell lines, as detected in metastasis, indicating an important role in the progression of PCa [73-76, 123-126]. 

Others tumor metastasis genes — Caveolin-1 (CAV1), H-Cadherin (CDH13), EPHA7, and S100A2 are often downregulated in prostate tumor tissues when compared to adjacent normal tissues due to methylation [47, 74, 76, 81, 85, 92, 127-134]. Gene-silencing of CAV1 and CDH13 is associated with clinical features of PCa progression [132, 135, 136]. S100A2 methylation was seen in 75% of cases of nonmalignant tissues and in 100% of cases of BPH [133]. 

## 4 DNA HYPOMETHYLATION

DNA methylation in mammalian genomes is a DNA preservation mechanism by which repetitive DNA, (~50% of genome’s content), is transcriptionally silenced to prevent their expression and function [137]. Demethylation of normally methylated DNA, also known as hypomethylation, can disrupt such a defense mechanism, leading to structural and functional alterations of the genome.

There are two types of hypomethylation, global or genomic, which refer to an overall decrease of 5-methylcytosine content in the genome, and localized or gene-specific hypomethylation, which refers to a decrease in cytosine methylation relative to the “normal” methylation level. The latter process affects specific regions of the genome, such as the promoter regions of proto-oncogenes or normally highly methylated sequences, such as repetitive sequences and oncogenes [138]. Both global hypomethylation and gene-specific hypomethylation have been implicated in human cancers (Table **2**). 

### 4.1 Global Hypomethylation

Global hypomethylation, relative to the normal situation in PCa, has been reported in a few cases [28, 85, 139-143]. Subsequently, more sensitive and precise analysis of genome-wide hypomethylation has been conducted. LINE-1 retrotransposon sequences constitute between 5-10% of human genome and are strongly methylated in somatic tissues [144]. With LINE-1 hypomethylation analysis, the frequencies of DNA hypomethylation in PCa are reported to be 7-53%, leading to the hypothesis of a relationship between LINE- 1 hypomethylation and GSTP1 hypermethylation, or alterations on chromosome 8 [85, 139, 140]. 

Genome-wide hypomethylation, which could lead to activation of previously silenced genes, is seen in some advanced, metastatic PCa [28]. Heparanase degrades heparin sulfate and has been implicated in tumor progression. Increased heparanase expression in PCa tissue has been reported to be caused by promoter hypomethylation with an up-regulation of the transcriptional factor, Early Growth Factor Response1 (EGFR1) [142]. Cytochrome P450 1B1 (CYP1B1), a member of the CYP superfamily that is overexpressed in PCa, is also regulated by a hypomethylation of its promoter/enhancer region [143]. 

### 4.2 Gene-Specific Hypomethylation

Genes from cancer cells but not from normal cells are substantially hypomethylated [145]. Moreover, when compared to adjacent normal tissues, most cancer tissues contain two hypomethylated *ras *oncogenes, c-Ha-*ras *and c-Ki-*ras *[146]. In the prostate, the Plasminogen Activator Urokinase (PLAU) gene is highly expressed in most PCa tissues [147] and invasive PCa cell lines [148]. The PLAU gene encodes urokinase plasminogen activator, a multifunctional protein that can promote tumor invasion and metastasis in several malignancies, including PCa. DNA methylation may also play a role in the regulation of the PLAU gene in PCa, with hypomethylation of the PLAU promoter being associated with its increased expression in hormone-independent PCa cells, higher invasive capacity *in vitro*, and increased tumorigenesis *in vivo*. However, in normal prostate epithelial cells and in hormone-dependent LNCaP cells, the PLAU promoter is methylated, resulting in lower expression of the gene [149]. Another hypomethylated PCa gene is the Cancer Associated Gene (CAGE), a novel cancer/testis antigen gene [150]. Hypomethylation of CAGE, which occurs in about 40% of PCa cases, is responsible for its exclusive expression in cancer tissues [151]. 

## 5 RACIAL DIFFERENCES AND DNA METHYLATION IN PROSTATE CANCER

PCa is associated with racial disparities. In 2007, PCa was responsible for 37% of all malignancies in black men in the US. The PCa incidence and mortality rate is approximately 60% and up to threefold higher, respectively, in African American (AA) men than in the Caucasians (CAs). This tendency has been validated for more than 20 years before or after the PSA era [152]. Similar incidence and mortality rates have been reported in black men of West African ancestry from the Caribbean and South America [153]. The underlying reasons for such disproportionate ethnic differences in PCa prognosis and mortality are unclear. In part, genuine racial differences in cancer genetics and biology, sociocultural differences and/or access to health care systems are responsible, but these factors do not totally explain the higher mortality rate in African Americans with PCa. Limited knowledge is available for inter-racial differences on gene-specific or genome-wide methylation or other epigenetic processes in normal individuals or patients with PCa. Kwabi-Addo *et al. *[154] examined the methylation levels of six genes (*GSTP1, AR, RARβ2, SPARC, TIMP3*, and *NKX2-540*), which have been previously shown to be hypermethylated in Caucasians with PCa or cell lines. They compared matched and PCa tissues from AAs and CAs who had comparable Gleason scores. They observed significant differences in the methylation levels in all genes, *except GSTP1*, in the AA samples in comparison with CA samples [154]. 

This observation is in agreement with work by Woodson *et al. *[155], which also demonstrated an increase but no significant difference in *GSTP1 *methylation in PCa tissues and benign prostatic hyperplasia samples from AAs compared to CAs. Also, it was demonstrated that *NKX2-5* and *TIMPC* genes were highly methylated in normal prostate tissue samples from AAs compared to CAs and that methylation of NKX2-5 increased with age in AAs. Thus, it was suggested that NKX2-5 may serve as a marker for PCa detection and increased sensitivity for detection PCa in AAs since the incidence of PCa increases dramatically with age. Although Kwabi-Addo *et al. *[154] observed the relationship between DNA methylation and PCa risk, they did not detect any significant association between DNA methylation based on race. Therefore, the utilization of these genes as “ethnic-sensitive” biomarkers for PCa is promising and can be further assessed with a larger PCa population size, as noted by the authors.

In addition to the aforementioned genes, the adhesion and signal transduction membrane protein CD44, which is associated with the progression of localized cancer to metastatic disease, has also been shown to be hypermethylated in AAs. Woodson *et al*. [155] examined the methylation of three genes involved in the progression of PCa, GSTP1, CD44, and E-Cadherin. As noted earlier, there was an increase but no significant difference in *GSTP1 *methylation, as well as no difference in the frequency between AAs and CAs. While E-Cadherin was not hypermethylated at all, hypermethylation of CD44 was observed among AAs with higher frequencies compared to CAs and correlated with tumor grade but not disease stage. However, Kito *et al.* reported a correlation between CD44 hypermethylation and disease stage in a Japanese men [156]. Although it is speculated that CD44 hypermethylation may have prognostic implications, its methylation and role in racial differences in PCa should continue to be explored in larger studies.

## 6 HISTONE MODIFICATIONS

In eukaryotes, genomic DNA is packaged with histone proteins into chromatin, compacting DNA some 10,000-fold. Such condensation of DNA provides a considerable obstacle to the nuclear machinery that drives processes such as replication, transcription or DNA repair. Importantly, the structure of chromatin dynamically changes, permitting localized decondensation and remodeling that facilitates the progress of nuclear machinery. 

The chromatin structure is regulated by a variety of post-translational modifications including DNA methylation, modification of histones, and ATP-dependent chromatin remodeling. Histones can be modified by several post-translational mechanisms including acetylation, methylation, phosphorylation, ubiquitination, sumoylation, or ribosylation of distinct amino acids, resulting in either the activation or suppression of gene expression [157-161]. 

A complete understanding of the precise molecular mechanisms by which the histone tail alterations influence DNA-histone interactions remains elusive. There are two main hypotheses on how histone modifications can affect chromosome function: 1) they may alter the electrostatic charge of the histone, resulting in a structural change in the histones or their binding to DNA; or 2) these modifications are binding sites for protein recognition motifs, such as the bromodomains or chromodomains, that recognize acetylated lysines or methylated lysines, respectively [162]. 

Enzymes that tightly control the balance of covalent histone modifications are histone acetyl transferases (HATs) and HDACs as well as histone methyltransferases (HMTs) and demethylases (HDM). These enzymes alter the configuration of the chromatin and regulate gene expression. Acetylation of lysine residues in histone tails by HATs unpacks the chromatin structure and renders the DNA accessible to transcription factors, thereby facilitating gene expression. The effects of HATs are counteracted by HDACs, which pack chromatin and repress gene transcription. In mammals, there are 18 HDACs which are subdivided into four distinct classes based on sequence homology to yeast HDACs and functional similarities. Class I HDACs (1, 2, 3, and 8) are primarily located in the nucleus and are ubiquitously expressed. Class II HDACs are divided into two subgroups: IIa HDACs (4, 5, 7, and 9) and IIb HDACs (6 and 10), which shuttle between the nucleus and cytoplasm and show a tissue-specific expression pattern [163, 164]. The NAD+-dependent enzymes of Class III HDACs (also named SIRTUINS) comprise seven members (SirT1– 7) and are ubiquitously expressed. Although most of these enzymes were shown to regulate histone acetylation, their distinct biological functions are largely due to the deacetylation of non-histone proteins such as transcription factors. 

Overall, post-translational modifications of histones create an epigenetic mechanism for the regulation of a variety of normal and disease-related processes, including cancers. Drugs affecting histone modifications already have been developed and have shown promising results in the treatment of different tumor types. 

## 7 INTERACTION BETWEEN DNA METHYLATION AND HISTONE MODIFICATION IN PROSTATE CANCER 

The silencing mechanism of DNA methylation is only part of a complex set of epigenetic regulatory processes. Another epigenetic change that is distinct from and yet linked to DNA methylation is the modification state of the surrounding histones in which the DNA is packaged. DNA and histones are linked functionally to control transcription and repair. It has been shown that methylated DNA recruits HDAC through methyl-DNA binding proteins (MBPs); consequently, DNA methylation/ histone deacetylation cross talk has been suggested to influence gene silencing [10, 12, 165-169]. 

The histone modifications exist in relative balance, maintained by competing enzymes that constantly work to place and remove the appropriate modifications. The signals that control these modifications are complex and can include DNA methylation and the binding of other protein co-factors [170]. In PCa, a number of *in vitro* studies provide evidence that promoter hypermethylation and histone deacetylation interact to maintain chromatin in its inactive state. These studies have shown that combined treatment with the histone deacetylase inhibitor, Trichostatin A, and demethylating agents 5-aza-cytidine or 5-aza-2’- deoxycytidine led to reversing epigenetic silencing of several genes. A loss of hypermethylation in the promoter and concomitant gene activation has been observed for a number of tumor-suppressor genes in various PCa cell lines. 

Transcriptional activation of a gene is facilitated by the addition of acetyl groups on the N-terminal lysines of histones to create an open or noncompact chromatin (euchromatin) conformation. The addition of acetyl groups on histones, primarily histones H2a, H3 and H4, is catalyzed by HATs. Increased HAT activities have myriad effects that may alter PCa growth in positive and negative ways. They can regulate transcriptional co-regulator proteins that bind to steroid receptors. For example, the HATs p300, PCAF and Tip60 up-regulate the expression or activation state of the AR which leads to an increase in the AR signaling in the absence of androgen [171, 172]. HATs also increase IGFBP-2 and p21 activation in PCa, slowing cell-cycle progression [4]; thus, HATs affect a range of cellular processes and as such they represent a putative target for therapy. 

## 8. EPIGENETIC CHANGES AND PROSTATE CANCER DIAGNOSIS, PROGNOSIS, AND TREATMENT

Prostate-specific antigen (PSA) is a less than-optimal tumor marker and cannot effectively differentiate between PCa and other conditions such as prostatitis or BPH. The false positive results lead to expensive and invasive critical investigations such as transrectal prostate biopsies. This provides the opportunity for researchers to identify more reliable potential epigenetic markers for PCa diagnosis, prognosis, and follow up treatment. 

### 8.1 Epigenetic Diagnostic Markers

Epigenetic markers, particularly aberrant DNA methylation, have the potential to be useful diagnostic tumor markers. Historically, there have been two general strategies for detecting DNA methylation changes at specific DNA sequences in cancer cells. The older approach exploits the use of methylation-sensitive restriction endonucleases, which cut recognition sites differently depending on whether 5-meCpG is present [173, 174]. The second major strategy for selective detection of genome sequences carrying methylation marks features DNA modification using sodium bisulfite, a procedure that promotes cytosine deamination to uracil but spares 5meC [175, 176]. Methylation markers have several advantages over the mutation-based genetic markers. Aberrant DNA methylations are more frequent than mutations and can be identified by genome-wide screening methodologies. DNA methylation markers potentially could be tested in tumor tissues and body fluids (e.g., serum, urine). The mthylation markers are simple in nature; with high sensitivity, these markers can be detected, either quantitatively or qualitatively, by available well-established techniques (e.g., PCR). Furthermore, the incidences of aberrant DNA methylation are higher than those of mutations and can be discovered by genome wide screening procedures [177]. 

One of the best characterized epigenetic markers in PCa is *GSTpi, *a gene that is hypermethylated and silenced in more than 90% of all PCa [178]. It is specific for PCa, although methylation of this gene is also found in proliferative inflammatory atrophy in the prostate, a histological entity that has been linked to PCa development [42]. It has also been reported that a methylation assays combined with histological analysis improves the diagnostic specificity [179]. Several groups examined body fluids containing prostate cells, including serum, urine and semen, for the presence of GST*pi *methylation in patients with cancer. The GST*pi *protein is clearly detectable in the urine of patients with PCa [180], especially after prostate biopsy. Analysis of prostatic secretions obtained from 100 patients at radical prostatectomy demonstrated 74% sensitivity, and a positive association between the degree of methylation at GST*pi *and the extent of the cancer [181]. *GSTpi* CpG island hypermethylation was found in 72% of serum samples from patients with PCa [182], and it was found to be a significant predictor of PSA recurrence in the cell-free serum of men with PCa [183]. 

Methylations of several other genes have been studied in PCa diagnosis including, RARβ, CD44, E-Cadherin (ECAD), RASSF1A, APC and tazarotene-induced gene 1 (T1G1) [75, 76]. Others studies have reported that the use of a panel of methylation markers including GSTP1 improves the diagnosis of PCa both in body fluids and tissues. Further studies are needed before these markers can be used as diagnostic markers in routine clinical practice. 

### 8.2 Epigenetic Prognostic Markers

One study demonstrated that GSTP1 hypermethylation is seen in 40% of pre-operative bone marrow aspirate in patients with advanced PCa [184]. Caveolin-1 (CAV1), E-Cadherin (CDH1), H-Cadherin (CDH13), EPHA7, and S100A2 are the tumor metastatic genes that are often more down-regulated in prostate tumor tissues than in adjacent normal tissues due to methylation [47, 74, 76, 81, 84, 85, 127-134]. Silencing of Gene CAV1, CDH1, and CDH13 is associated with clinical features of PCa [132, 135, 136]. These data suggest that the methylation status of CAV1 and CDH1 is not only a potential biomarker for PCa, but may also be a predictive marker of disease outcome [135]. Genes such as CD44 and T1G1 may exhibit specific methylation in high-risk and metastatic tumors and could be used in molecular staging and as predictors of disease progression [78]. Prostate cancers with a high Gleason score are correlated with a higher degree of methylation of many genes, such as RARβ, RASSF1A, and GSTP1 [185]. 

### 8.3 Epigenetic Changes as Therapeutic Targets

Epigenetic information is heritable, but has plasticity. Dynamic erasure and writing of epigenetic information take place in specific genes during embryonic development [186]. This makes it possible to modify unwanted epigenetic changes. So far, several classes of drugs, including inhibitors of DNMTs and HDACs, are known to modify epigenetic information in a manner that is not specific to genes. Currently, there are several drugs that are at varying different stages of development. They can be broadly classified in two groups: (i) DNMT inhibitors and (ii) HDAC inhibitors. Some of the drugs are in both groups. 

#### 8.3.1 DNMT Inhibitors

DNMTs, especially DNMT1, play important roles in maintaining CpG methylation [182], and their inhibitors are known to induce hypomethylation. In particular, an inhibitor of DNMT1, 5 aza-C, and 5-aza-20-deoxycytidine (5-azadC; Decitabine), are two closely related drugs used experimentally to inhibit DNA methylation *in vitro* and have been shown to re-activate numerous methylation-silenced genes, such as GSTP1 [183] and RARβ2 [72]. 5-aza-C and 5-aza-dC are cytosine analogues that become incorporated into DNA and trap DNMT1 during replication, leading to the synthesis of nascent DNA in the absence of DNMT activity, resulting in DNA demethylation [187-190]. Myelo-suppression is a major adverse effect, but the treatment is well tolerated. 5-aza-dC has been recently approved by FDA for clinical use in certain hematological conditions. Another drug in the same group, Zebularine can be administered orally or intraperitoneally, but has to be given in high doses; however, it is chemically stable and has low toxicity [191]. 

Among the chemicals already in clinical use or in food, procainamide, procaine and epigallocatechin-3-gallate (EGCG) have also shown demethylating activity [192-194]. Considering that some aberrant DNA methylation is present in the early stages of carcinogenesis, it is possible that such demethylating agents may be useful for cancer prevention. MG98 is a phosphorothioate antisense oligodeoxynucleotide, and is a specific inhibitor of DNA methyltransferase mRNA; this drug is also used for demethylation [177]. 

#### 8.3.2 Histone Deacetylase Inhibitors

In recent years, it became evident that HDACs are promising therapeutic targets with the potential to reverse aberrant epigenetic states associated with cancer. Various studies in cancer cell lines and tumor tissues revealed changes in the acetylation levels and the expression of the HDAC enzymes [195]. Increased HDAC activity in prostate tumors provides another avenue for therapeutic inhibition. A variety of natural products exhibit HDAC-inhibitory activity. Commonly used HDAC inhibitors that are being tested include trichostatin A (TSA), Suberoylanilide hydroxamic acid (SAHA) and valproic acid [5]. These drugs appear *in vitro* to also increase the efficacy of radiation therapy [196] and they have anti-angiogenesis function [197]. SAHA, a class I HDAC inhibitor, significantly represses the growth of LNCaP hormone sensitive prostate tumors in nude mice (97% inhibition vs untreated controls) [198]. Trichostatin A (TSA), leads to hyperacetylation of histones and cell cycle inhibitors, such as p21WAF1 [199]. The class I inhibitor and benzamide derivative, MS-275 increases xenograft radiosensitivity and inhibits prostate tumor growth .The HDAC*i, *LBH589 targets class IIa HDACs and decreases angiogenesis in PC-3 xenograft tumors [200]. Various HDAC inhibitors have been developed for therapeutic purposes, and tumor cells are known to show higher sensitivity than normal cells for unidentified mechanisms. HDAC inhibitors are also reported to be effective even in non-proliferating tumor cells *in vitro* [201]. 

The combination of HDAC and DNMT inhibitors has synergistic effect in the reactivation of silenced gene [5]. Another interesting possibility is the combination of epigenetic drugs and conventional anti androgens and chemotherapeutic agents. It should be cautioned that the epigenetic drugs currently lack gene specificity and some of them are associated with significant toxicity. Hence, efforts are being made to develop gene specific epigenetic drugs [177]. 

## 9 METHODS FOR DETECTION OF DNA METHYLATION

**Fig. (3):**
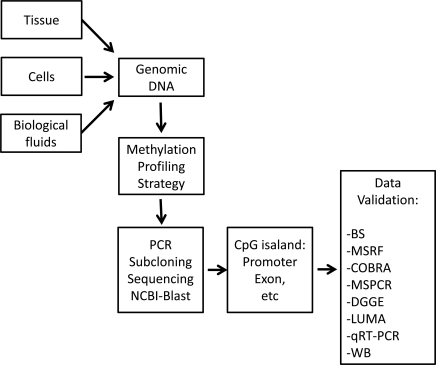
**Step-by-step approach from the discovery to validation of the CpG methylation.** Genomic DNA could be extracted from different types of biological specimens. Based on the investigators need methylation study could begin using a variety of methylation profiling tools and could be followed by PCR and subcloning into a vector, sequencing, aligning into NCBI-Blast data base. After proper identification of the gene or sequence of interest or candidate CpG islands, and designing specific primers, final data validation assays is necessary to confirm the relationship between methylation level and gene or protein expression. This will be performed by different methods listed in the flow chart. BS, Bisulfite sequencing; MSRF, methylation-sensitive restriction fingerprinting; COBRA, Combined bisulfite restriction analysis; MSPCR, methylation-sensitive PCR; DGGE, denaturing gel electrophoresis; LUMA, luminometric methylation assay; qRT-PCR, quantitative real-time PCR; WB, western blotting.

A wide range of methods are available to either quantitatively or qualitatively to find methylated changes in genomic DNA. Based on the type of technique used, several major groups of detection methods are described here (Fig. **3** and Table **3**).

### 9.1 Bisulfite Sequencing

This is the most widely used method to examine the methylation status of individual cytosines within any amplified portion of a gene [202]. It is based on a simple principle; the ability of sodium bisulfite to deaminate un-methylated cytosine residues into uracil in genomic DNA. Following PCR amplification, the uracils will be amplified as thymines. Cloning and sequencing of the DNA fragments will identify the methylated cytosine in CG-islands.

### 9.2 Methylation-Sensitive Restriction Fingerprinting (MSRF) 

In this technique, methylated DNA will be subjected to restriction enzymes *Mse*I and/or *Bst*UI digestion followed by PCR amplification of DNA [203]. Extracted genomic DNA will be digested first by *Mse*I enzyme which recognizes the TTAA restriction site, which rarely exists in CG-rich areas. Therefore, DNA is cleaved into small pieces with the intact CG-regions. Subsequently, a fraction of the *Mse*I-digested DNA will undergo a second digestion by a methylation-resistant restriction enzyme, *Bst*UI. This enzyme is able to cut un-methylated CGCG segments of the DNA which exists in >80% of CG-rich area. The remaining small undigested fragments of DNA of *Bst*UI digested and *Mse*I-only digested DNAs will be amplified using random-primer PCR and compared. 

### 9.3 Chromatin Immunoprecipitation on DNA Microarray (ChIP-chip)

In this method, live cells will be fixed by formaldehyde crosslinking. After isolating intact genomic DNA with transcription factors, antibodies specific to transcription factors of interest will be used to immuoprecipitate the chromatin-protein complex of interest. Next, DNA will be extracted by reversing crosslinking, purified, and chromatin amplicons will be generated by PCR. Amplicons will be labeled with Cy5, and Cy3will be used to label the input reference amplicons. These labeled probes will be applied on a CG array for hybridization and data will be analyzed for the specific interaction between a unique gene-specific CpG site and a specific transcription factor [204].

### 9.4 Luminometric Methylation Assay for Global DNA Methylation Analysis

Luminometric methylation assay (LUMA) is based on DNA cleavage by a combinations of restriction enzymes, *Eco*RI/*Msp*I or *Eco*RI/*Hpa*II, which leaves the TTAA (*Eco*RI) and methylation-sensitive CG (*Hpa*II) or methylation-resistant *Msp*I overhangs. Bioluminometric polymerase extension will be used to quantify the extent of restriction cleavage by Pyrosequencing. Following the DNA cleavage, the methylation status will be determined by PCR or Southern blotting. The extent of CpG sites methylation could be accurately and quickly determined by Pyrosequencing, which dispenses labeled nucleotides and primers and gel-electrophoresis. LUMA has been used to evaluate the level of methylated cytosine of the whole genome, more frequently to study specific CG islands, to determine the association between changes in methylation status, and diseases, and as diagnostic or prognostic markers of cancers [205-209]. 

### 9.5 Matrix-Assisted Laser Desorption/Ionization Time-of-Flight Mass Spectrometry (MALDI-TOF-MS)

This is a powerful technique to analyze DNA methyla-tion [210, 211]. A variety of mass spectrometry approaches have been developed to measure DNA methylation levels such as rapid screening of single nucleotide polymorphisms and quantitative allele studies. In addition, it has been used to assess nucleotide digestion and DNA sequencing. It involves bisulfite conversion of genomic DNA followed by DNA sequencing, which leads to accurate determination of the levels of genomic DNA methylation. MALDI-TOF-MS can measure the gene-specific or genome-wide content of methylated cytosine in a high-throughput manner with the advantage of high speed, accuracy, and automation. 

### 9.6 Illumina Human Methylation 27/450 Beadchip Array

Genome-wide promoter methylation could be evaluated by Illumina Infinium Human Methylation 27/450 Bead array. This analysis was made at the Bioinformatics and Expression Analysis core facility at the Karolinska Institute. The EZ DNA methylation kit (Zymo Research, Orange, CA) could be used for bisulfite conversion of genomic DNA, and the remaining assay steps could be performed using Illumina-supplied reagents and conditions. The Illumina Infinium II Bead Array uses allele-specific annealing to either methylation-specific probes or non-methylation probes to detect the methylation grade of 27,578 individual CpG sites spread across the promoter regions of 14,495 genes [212, 213]. The HumanMethylation450K BeadChip includes 90% of the content contained on the HumanMethylation27 BeadChip, is designed in the same 12-sample per array format. Its unique feature combines unprecedented quality of coverage and study design flexibility in running genome-scale methylation screens.

### 9.7 Methylation Analysis by Denaturing Gradient Gel Electrophoresis (DGGE)

PCR products of bisulfate reacted different gene promoter regions could be analyzed using denaturing gradient gel electrophoresis (DGGE). In brief, the PCR products are loaded on a 10–70% denaturant gradient gel together with a fully methylated control (e.g., *in vitro* methylated DNA) and an unmethylated control (e.g., peripheral blood lymphocytes). After electrophoresis, gels will be stained in Tris/EDTA buffer containing ethidium bromide and photographed under ultraviolet transillumi-nation. Samples are scored as methylated when bands or smears are present on the gels in the area below the band corresponding to unmethylated DNA [214, 215]. 

## 10 DNA METHYLATION DATA BASE AND COMPUTATIONAL ANALYTIC TOOLS

During the last decade, epigenetics emerged as a vibrating field in biological sciences and specifically cancer research that led to significant amount of experimental data. Among all others, DNA methylation is the most investigated epigenetic alterations. These developments necessitate the creation of multi-task or subject-specific databases and more and more high-throughput computational methodologies capable of handling the enormous experimental data that could be tailored for discovering the most relevant biological information. Based on the epigenetic profile and in translational settings, this knowledge could be used for risk assessment, screening, prognostic or diagnostic classification, and the development of novel therapeutic strategies toward cancers.

### 10.1 Databases for CpG Detection Sites

Detection of CG dinucleotides and CpG islands are central to DNA-methylation research. Based on cytosine and guanine frequencies, the CG dinucleotide occurrence in the human genome is considerably less than what is expected for all the methylated cytosine in the entire genome. The CpG islands are defined based on a few default or most common parameters that includes a minimal length of approximately 200 bp, a CG content of approximately 55%, and the observed/expected ratio of 0.6 for CG dinuleotides. Based on these and other parameters, several data bases are available to identify, plot, and report CpG islands in selected DNA fragments, such as CpGReport, NewCpGReport, CpGplot, and isochore, which are part of the European Molecular Biology Open Software Suite (EMBOSS). 

### 10.2 Databases for Determination of Transcription and Translation Initiation Site

Methylation silencing of DNA occurs most frequently in CpG islands within the promoter region of genes. Additionally, the CG dinuleotides of the first exon and intron also have important biological significance in terms of alternate splicing, multiple transcripts, and protein isoforms and functions. Therefore, determination of the transcription start site and translation start site are required for the DNA-methylation analysis of genes. The functional identity of the predicted transcription or translational start site (TSS) could be experimentally validated. Several databases provide this information including the DataBase of Human Transcription Start Sites (DBTSS), Eukaryotic Promoter Database (EPD), and Ensembl. DBTSS provides the location of TSS, retrieval of promoter sequence, extraction of cDNA sequences upstream or downstream to TSS [221]. DBTSS is based on 400,225 and 580,209 human and mouse full-length cDNA sequences and contains exact information on the genomic positions of the TSSs and the adjacent promoters for 8,793 and 6,875 human and mouse genes, respectively. The EPD is an annotated non-redundant collection of eukaryotic POL II promoters for which the TSS has been experimentally determined [222]. Available information on this site is either extracted from scientific literature or obtained by *in silico* primer-extension method. Access to promoter sequences is provided by pointers to positions in nucleotide sequence entries. The annotation part of an entry includes description of the initiation site mapping data, cross-references to other databases, and bibliographic references. EPD is structured in a way that facilitates dynamic extraction of biologically meaningful promoter subsets for comparative sequence analysis. Ensembl is established as a joint initiative between EMBL-EBI and the Wellcome Trust Sanger Institute to develop a software system for automatic annotation on promoters, TSSs, exonic and intronic positions of the eukaryotic genomes in more than 50 species (e.g., human, rat, mouse, zebrafish) [223].

### 10.3 Analytical Tools for Experimental Design of DNA Methylation

Epigenetics research has benefited vastly from molecular biology methodologies including PCR, DNA sequencing, and restriction enzyme digestion and analysis. Most commonly used modified versions of these techniques are bisulfite conversion and sequencing, methylation-specific PCR, and combined bisulfite restriction analysis (COBRA). DNA treatment with bisulfite leads to deamination of unmethylated cytosine residues into uracil. Therefore, bisulfite treatment is the introductory to determine the overall CpG methylation rate or status of any suspected DNA fragment. *In silico* conversion of DNA fragments is made possible by Web-based software such as BiQ Analyzer that provides bisulfite conversion in addition to reversed sequence and retrieval of the complementary strand [224]. Following bisulfite conversion, discrimination between methylated and unmethylated cytosine residues could be accomplished by designing the methylation-specific or bisulfite sequencing primers using software such as MethPrimer, MethMarker, or MethBLAST. MethPrimer detects CpG-specific parameters and islands and is essentially based on the primer3 algorithms for designing primers [225, 226]. In addition, MethMarker used as epigenetic primer design software can be used for epigenetic biomarker optimization and the design of assays such as bisulfite single-nucleotide primer extension (SNuPE), bisulfite pyrosequencing, MSP, methylation analysis by DNA immunoprecipitation (MeDIP)-qPCR. MethBLAST software uses a BLAST search on a bisulfite converted product of methylated and unmethylated cytosine residues of the database sequences. It can identify amplicons in the converted genome, and determine the specificity of generated MSP or bisulfite sequencing primers. COBRA is a DNA-methylation analysis strategy that uses methylation-sensitive restriction enzymes. Consequently, the length of the resulting fragments will be used to determine the methylation state and site-specificity [227]. The restriction enzyme catalogue of New England Biolab (NEB), NEB database REBASE [228] and MethMarker can provide a comprehensive list of methylation-sensitive restriction enzymes to be used in setting up a COBRA experiment.

### 10.4 DNA Methylation Database 

Several databases provide general or specific information for epigenetic research. Methdb is a general purpose methylation database listing the results of methylation experiments ranging from overall genome methylation status to site-specific measurements; it covers several species and a wide range of methodologies and sample types for broad and general methylation studies [229]. MethprimerDB is a database linked with MethDB and contains the published primers that could be used as positive control in experimental settings [230]. This database contains probe and primers to test methylation in techniques such as COBRA, bisulfite-PCR-SSCP, bisulfite sequencing, MSP, and Ms-SNuPE. 

## 11 FUTURE CHALLENGES AND CONCLUDING REMARKS

PCa epigenome is a prototypic catastrophic model of epigenetic alterations in tumorigenesis and disease progression. Alterations in DNA methylation status in tumor-suppressor genes, oncogenes, and other regulatory or structural genes are the most frequent epigenetic processes identified in PCa. The importance of promoter hypermethylation in silencing critical tumor-suppressors (e.g., *APC, RASSF1*) in PCa is well documented. Global hypomethylation has been identified in primary and metastatic PCa. Genome-wide hypomethylation has been associated with high grade and advanced stage tumors. Overall, the combination of promoter hypermethylation and genome-wide hypomethylation might act favorably in prostate carcinogenesis and progression. The epigenome offers a number of promising prognostic, diagnostic, and therapeutic targets for PCa. However, several obstacles need to be addressed. The plasticity of epigenetic processes might open therapeutic opportunities, but at the same time the reversibility of these processes after treatment is of great concern. In addition, non-specific targeting of epigenetic process by available drugs might lead to short-term or long-term undesirable side effects. This could be resolved significantly by developing gene-specific sustainable demethylation approaches. It is not surprising that the field of PCa research has focused on identifying “epigenetic signature” that could be used for prevention, early detection, progression, or distinction between organ-confined indolent and aggressive disease. At least for early detection, aberrant promoter methylation appears to be the most promising early indicator of PCa. Overall, epigenetics is a fascinating and expanding field in modern biology that has turned scientific, clinical, and public attention to its practical applications for cancer prevention, detection, and therapy. 

## CONFLICT OF INTEREST

	None.

## Figures and Tables

**Table 1:** Genes Frequently Hypermethylated in Prostate Cancer

Gene	Chr^a^	Role/Function	Hyper methylation	Prostate cancer		Cell lines^d^	Ref.
				Primary^b^	Met^c^		
Glutathione S-transferase Pi (GSTP1)	11q13	Intracellular detoxification	70% - 100%	+		+	[50]
Glutathione-S-transferase (GSTM1)	1p13	Intracellular detoxification	58%	+			[178]
O-6-Methylguanine DNA-Methyltransferase (MGMT)	10q26	Remove alkyl adducts from O^6^-guanine	76%	+		+	[44]
Retinoic Acid Receptor beta (RAR β)	3p24	Tumor suppressor	84%	+			[68]
Androgen Receptor (AR)	Xq12	Hormone regulation	39%	+			[216]
Estrogen Receptor Alpha (ERα)	6q25	Hormone regulation	90-95%	+			[56]
Estrogen Receptor beta (ERβ)	14q23	Hormone regulation	79-100%	+			[56]
Ras association domain family 1A (RASSF1A)	3p21	Tumor suppressor:cell growth	53-79%			+	[81]
Death Associated Protein Kinase 1 (DAPK1)	9q34	Regulator of cell growth	36%	+			[46]
Endothelin Receptor B (ENDRB)	13q22	Tumor suppressor	45-72%	+	+	+	[81]
E-Cadherin (CDH1)	16q22	Tumor suppressor: invasion and metastasis	61-72%	+			[76]
Cyclin-dependent kinase inhibitor2A/p16 (CDKN2A/p16)	9p21	Tumor suppressor	66%	+	+		[217]
Cyclin-dependent kinase inhibitor1C/p57 (CDKN1C/p57)	11p15	Tumor suppressor	56%			+	[218]
Cyclin-dependent kinase inhibitor1B/p27 (CDKN1B/p27)	12p13	Tumor suppressor	6%	+			[45]
Cyclin-dependent kinase inhibitor1A/p21 (CDKN1A/p21)	6p21	Tumor suppressor	6%	+			[45]
Tissue Inhibitors of Metalloproteinase-2 (TIMP-2)	17q25	Tumor suppressor	78.5%			+	[219]

a Chr: Chromosomal location.

b Primary prostate cancers.

c Mets: Metastatic prostate cancers.

d Prostate cancer cell lines.

**Table 2:** Genes Frequently Hypomethylated in Prostate Cancer

Gene	Chr^a^	Role/Function	Hypo methylation	Prostate cancer		Cell lines^d^	Ref. #
				Primary^b^	Mets^c^		
Urokinase Plasminogen Activator (uPA)	10q24	Tumor invasion and metastasis	75-96.9%	+		+	[220]
Heparanase (HPSE)	4q21	Tumor invasion and metastasis	8.5 -30%	+		+	[142]
Cancer/testis Antigen Gene (CAGE)	6p24	Cell cycle control: cellular proliferation	34%			+	[151]
Cytochrome P4501B1(CYP1B1)	2p21	Hydroxylation of estrogens and activation of carcinogens	5 17%	+		+	[143]

a Chr: Chromosomal location.

b Primary prostate cancers.

c Mets: Metastatic prostate cancer.

d Prostate cancer cell lines.

**Table 3:** Available Techniques for Detection of DNA Methylation

Technique	Gene-Specific or Genome-wide	Sample (µg)	Advantage and limitations	Ref.
Bisulfite Sequencing	Gene-specific Applicable to genome-wide	0.2-0.5	Gene-specific sequence, Simple set up, Most economic, Prone to false positive data, Needs additional confirmatory step, Difficulty in primer design, DNA degradation during bisulfite treatment	[202]
MSRF	Genome-wide	0.1-1.0	Simple set up suitable for novel genes screening, Special set up for gel electrophoresis	[203]
ChIP-chip	Genome-wide	1.0-10	High-throughput, Not common, Platform- specific	[204]
LUMA	Both	0.2-0.5	High-throughput, Relatively expensive, Limited sequence size	[205-209]
MALDI-TOF-MS	Gene-specific	0.01-1.0	High-throughput, Mostly for specific genes of interest	[210, 211]
Illumina Methylation Beadchip Array	Genome-wide	0.2-0.5	High-throughput, High accuracy, Relatively expensive, Platform-specific	[12, 213]
DGGE	Gene-specific	0.5-1.0	Very sensitive to variation in DNA sequence, Simultaneous analysis of multiple samples possible, Time-consuming, No method for automated analyses currently available	[214, 215]

MSRF: Methylation-Sensitive Restriction Fingerprinting. ChIP-ChIP: Chromatin Immunoprecipitation on DNA Microarray. LUMA: Luminometric methylation assayMALDI-TOF-MS. MALDI-TOF-MS: Matrix-Assisted Laser Desorption/Ionization Time-of-Flight Mass Spectrometry. DGGE: Denaturing Gradient Gel Electrophoresis.
